# Clinical Reflections

**Published:** 1883-11

**Authors:** Ad. Lippe

**Affiliations:** Philadelphia


### CLINICAL REFLECTIONS.

AD. LIPPE, M. D., PHILADELPHIA.

Mr. W., age 64 years, had previously enjoyed general good health; had been suffering for some ten days with nightly pains in the liver, accompanied by violent vomiting, first of water and finally of bile; he had taken by advice of a homœopathic physician Nux v. and Pulsatilla, with occasional relief. He grew worse and weaker. I found him on the 1st of September in bed after a very bad night. Color of the face yellow, painful soreness of the liver, constipation, urine normal, had vomited all night and complained of continued nausea; tongue swollen and coated yellowish white. Pulse, sixty beats per minute. Aversion to food and entire thirstlessness. He received one dose of Digitalis$^{cm}$ (Fincke) at 11 A. M. When he was again visited, twenty-four hours later, he was found much better; he had not again vomited, nausea had ceased, had only slight soreness of the liver. Pulse, seventy-two per minute; tongue smaller and almost clean. Received no more medicine, and called at my office five days later ready to go to the country.

Case II.—Mr. T., 32 years old, also very rarely indisposed, was visited September 1st. Complains of very acute pain in the liver, with vomiting, first of water and finally of bile; much worse at night. Yellow color of the face; urine dark and abundant; thirst for cold water; tongue swollen, showing on the edges the indentations of the teeth. Aversion to food. Pulse, eighty-four per minute. Gave one dose of Mercurius vivus$^{cm}$ (Fincke), much improved for forty-eight hours, when another attack of increased pain in the liver and vomiting as before returned; repeated Merc. viv. Two days later the tongue was better, the pain in the liver much less, but he had a chill about 8 P. M., followed by fever and perspiration; two days later he had another severe chill, preceded by much thirst for cold water; the thirst continues during the fever and during the perspiration; entire want of appetite during the apyrexia; tongue better but still swollen. The chills and fever and perspiration returned every other day at irregular hours, but became each time less severe; the urine becomes now of normal color, and the appetite gradually returns; as his condition, in every respect improved, he received no more medicine; after twenty-one days the chills ceased, but seven days later he had another slight chill, the tongue remained swollen, the color of the face normal; he now received another dose Merc. viv.$^{cm}$ (Fincke), and has been able to go out since; feels strong, and with the exception of a few very slight fever attacks feels better, and a fortnight after taking the last dose of Merc. viv. he reports himself well.

Case III.—Will be fully reported in the course of time; for the present we only give a partial sketch, as it was only a case of vomiting.

A lady, 54 years old, lost her husband twenty-three years ago at sea, he falling overboard from a steamer. For twenty-two years she was confined to her bed, and during these twenty-two years she did not retain any food. Her appetite was ravenous, she devoured her dinner especially because she was so very hungry, but as soon as the stomach was filled she had to throw the food up again, at times with much suffering and always followed by intense debility. During these twentytwo years she had the advice of the best allopathic physicians, who did by no means agree on a diagnosis, but they all agreed that the only way life could be prolonged was by absolute and entire rest. Every remedy she had taken made her much worse, and for three years she had given up all medicines, and had taken for an attack of cholera morbus she suffered from in the last fall, for one week large and repeated doses of various preparations of Opium, and while they caused much suffering, much delirium, and utter sleeplessness, she finally again rallied. My first visit was made in the beginning of June. The patient was terribly emaciated, of course, but her brilliant intellect was not in the least impaired. As it was impossible for me to detect any spinal disease, which some very learned physicians of the scientific school declared did exist, I ordered her to take all her meals while sitting up at table, which she has done ever since. A dose of Nux vom. was first administered, causing no effect whatever; seven days later, and after a very careful examination of the materia medica, one dose of Ferr. met.$^{cm}$ (Fincke) was given before bedtime. There was no effect discernible for three days. On the fourth and fifth day after taking Ferr. her bowels became very loose, but as she had no pain with these more than usually loose and frequent stools no medicine was given. On the sixth, seventh, and eighth days she vomited less and lost that intensely ravenous desire for food; there had been no change of food made, with the exception of the administration of iced champagne by the teaspoonful during her dinner. On the ninth day she ceased to vomit, and improved day by day for two months; was able to sit up for ten hours a day, and walked over the second floor of her cottage with facility. After this gradual improvement she was attacked with a similar attack of cholera morbus of which she had suffered about a year ago. The attack began early in the morning, continued during the forenoon, and wore off in the afternoon. On the second day of the attack she received one dose of Sulphur$^{21m}$ (F. C.), and gradually recovered. The opiates taken for the similar attack a year ago caused absolute sleeplessness and general wretchedness, but now after she took this single dose of Sulphur she very soon fell into a sound, refreshing sleep for three hours, which occurrence the observing healer values as a certain sign that the remedy was homœopathic to the case and is sure to strongly affect the case. After the diarrhœa had entirely ceased, after a few days, the food was again rejected, although the appetite had now become normal. Ferrum met. was again given without causing any improvement; she complained of great fullness after eating, even if she did not give up the food. Ignatia was now given, but she continued to give up the food frequently; she also began to increase considerably in size in the abdomen and had in it a great sensation of heaviness; there was no doubt that there was an accumulation of water in the abdomen. Gave her a dose of Sepia$^{cm}$ (Fincke), which caused her to pass a great quantity of urine for about a week; the swelling disappeared and so did the sensation of heaviness. After all the symptoms had subsided, a dose of Ferr. muriaticum$^{cm}$ (Fk.) was given; it did cause the same loose stools as Ferr. met. did, and the food is again retained.

*Comments.*—The first two cases had so many symptoms in common that it might very easily have led one to believe they required the same remedy. The congestion of the liver seemed in both cases, coming under treatment on the same day, to be the primary cause of the vomiting and of the pains; in both cases the tongue was swollen, the color of the face yellow, the aggravation was at night, but there was in the first case thirstlessness, in the second case much thirst; in the first case a slow pulse, in the second a rapid pulse. The first case was promptly improved under Digitalis, which was the similar remedy, and it is remarkable that some six weeks later a sudden attack of giddiness with exactly the same vomiting, a yellow complexion, and swollen tongue returned, and again, under the care of Dr. Walter M. James, Digitalis promptly relieved the patient. The second case was caused by malaria, and while it first appeared in a masked form it soon developed itself, and was promptly cured by the remedy so clearly indicated at the first appearance of the disorder; it was repeated at very long intervals when the effects of the previous dose seemed to be exhausted.

The third case is by far the most remarkable case of long-protracted sickness that ever came under my care. At a later date, if the now apparent improvement continues, this case will be fully reported. There are many physiological and pathological questions to be solved; they will find their solution through the observations made of the effects of the homœopathic methods of cure promulgated by Hahnemann, and even now this desperate case already shows how very excellent and how reliable are the rules Hahnemann taught us to follow, if we desired to apply the *only law of cure* existing. There have been new laws discovered by professing homœopathicians. We have been admonished to "progress and investigate," and we have even had an illustration of this new method of cure: a case was reported by the illustrious inventor of our new but differing laws which for *three long years* progressively grew worse, and was finally cured by a single dose of Pyrogen before bedtime. The remedy claims to have been homœopathized by means of a "Potentizer." If that case is truly related, then it would become us to apologize for not having progressed without reflection. At that rate, the disease (reported in case III) being of seven times as long a standing as the one *cured* in eight hours by a homœopathized remedy, should have been cured in fifty-six hours. Even with an ardent desire to test the efficacy of this newly discovered method of cure, it would have been extremely difficult to find the remedy in either of the three cases here reported. Was there in any of these cases to be found a morbific product of the disease? If we reflect and first find the morbific product of the disease, we might then homœopathize such a product, and having done so progress and test the applicability of the new law and the new methods; the difficulties we apparently first encounter, when we unwittingly progress without reflection, would be the discovery of "a disease," yet the true healer, we mean the homœopathician, *never* treats diseases by name. The discoverers of the new law of cure will probably be able to state under each name of a disease the "product of it," and as upon reflection we predict that the product of diseases treated as such by name will, in the large majority of cases, be—a funeral! Would it not be consistent with this huge heresy to submit the whole funeral, flower offerings included, to a newly discovered homœopathization—which new phrase we now use, having offended the highly sensitive friends of heresy by calling the new process of making an improved remedy even a nosode, a homœopathic remedy, without proving it, but by subjecting it to "a bottlewashing process"? Sensitive people who do not hesitate to offend common sense must be treated tenderly, and as they say that *bottlewashing* is an ungentlemanly and vulgar phrase to express the absurd assertion made, that a remedy, even unproved one, even a nosode, a *supposed* product of a disease, becomes a truly homœopathic remedy for the disease itself by submitting it to a process of "potentization," or fluction potentization, we shall in future restrain ourselves from calling this process by *that name* (bottlewashing), but shall put on kid gloves and call the newly promulgated heresy by which said perverters of common-sense Homœopathy declare that *any* substance becomes a homœopathic curative agent, even if never proved, etc., by potentization—call it *homœopathization.* It is very singular that the discoverer of a new law should address himself to an association he joined for the avowed purpose of eliminating from the Institute errors and departures that had gradually crept in, under the plea of freedom of medical opinion and action. It is still more singular that this International Hahnemannian Association, now in existence for over three years, has never yet made the slightest attempt to *reform* the Institute, but, on the contrary, *sanctions* the introduction, by one of its members, of a vastly viler heresy than the Institute ever presented, or, under the plea of freedom of medical opinion and action, allowed it to remain unnoticed. But here are historical facts to show the correctness of our assertions. Nothing has been done to vindicate the publicly expressed promises of eliminating from the Institute departures which have crept into that organization. A worse heresy than ever before offered is now foisted on the reformatory organization, and it is charged, with good reasons, that the I. H. A., as now represented by its officers and its executive committee, not only does not censure the member who so boldly claims to have found new laws and new methods, diametrically opposed to the law of the similars and Hahnemann's method of applying it for the use of the sick, aided by his master work, the *Materia Medica Pura*, but it has become an historical fact that this member, advocating the progress into the worst heresy ever offered the profession, is *sustained.* This new progressive departure from Hahnemann's methods *may(!)* lead to an easier manner of curing the sick, but as it is not Homœopathy, it may be well to first investigate its claims before we advance to adopt it and commit ourselves in sustaining a heresy. It is our intention to further dwell on this subject (the new heresy) in a paper now in preparation on the "Past, Present and Future of Homœopathy."

